# Detection of *Mycobacterium avium* subsp. *paratuberculosis* in reproductive tissue and semen of naturally infected rams

**DOI:** 10.21451/1984-3143-AR2018-0147

**Published:** 2019-11-18

**Authors:** José Vicente Velázquez-Morales, Marco Antonio Santillán-Flores, Jaime Gallegos-Sánchez, Juan Manuel Cuca-García, María del Carmen Navarro-Maldonado, Reyna Isabel Rojas-Martínez, César Cortez-Romero

**Affiliations:** 1 Colegio de Postgraduados, Programa de Ganadería, Montecillo, Texcoco, México; 2 Instituto Nacional de Investigaciones Forestales, Agrícolas y Pecuarias, Centro Nacional de Investigación Disciplinaria en Microbiología Animal, Ciudad de México, México; 3 Universidad Autónoma Metropolitana Unidad Iztapalapa, Departamento de Biología de la Reproducción, División de Ciencias Biológicas y de la Salud, Ciudad de México, México; 4 Colegio de Postgraduados, Ciencia Animal, Salinas de Hidalgo, San Luis Potosí, México

**Keywords:** *Mycobacterium avium*, *paratuberculosis*, tissues, semen, Pelibuey ram

## Abstract

*Mycobacterium avium* subsp. *paratuberculosis* (MAP) is the causative agent of paratuberculosis (PTB), disease that causes a syndrome of bad nutrient absorption, weight loss and eventually death. The intestine is the main target organ where the infection develops; however, there is evidence of infection by MAP in extra-intestine sites of sheep, including mesenteric nodes and semen. The aim of the study was to identify the presence of MAP in reproductive tissue and semen of infected Pelibuey rams in clinical state of PTB. Seven rams were used in clinical PTB state and a non-infected ram by MAP of the Pelibuey breed, confirmed by serology, nPCR and bacteriological culture, with average weight and age of 57.23 ± 1.73 kg and 2.91 ± 0.17 years, respectively. The presence of MAP was identified in different tissue samples: spleen (1/7, 14.3% and 2/7, 28.6%), small intestine (3/7, 42.9% and 4/7, 57.1%) and mesenteric lymph nodes (3/7, 42.9% and 3/7, 42.9%), with nPCR and culture, respectively. It was also identified in epididymis tissue (1/7, 14.3%), Cowper gland (2/7, 28.6%) and prostate (1/7, 14.3%), using nPCR, although without detection in culture. It was identified in testicular tissue in 42.8% (3/7; culture or nPCR technique), but in 28.6% (2/7) with both techniques. Finally, the presence of MAP was identified in 42.9% (3/7) of semen samples with nPCR; however, it was not detected through culture. In conclusion, the presence of MAP was identified in lymphatic, digestive tissue, and semen; the presence of MAP was reported for the first time in epididymis, Cowper gland, prostate and testicles of infected Pelibuey rams.

## Introduction

PTB or Jhone disease is chronic consumptive and incurable, caused by MAP; intracellular mycobacteria of slow growth, Gram positive, anaerobic, which has 14 to 18 copies of an insertion element called IS900 in its genotype (Ayele et al., 2001, 2004). It is a disease typical of ruminants (García and Shalloo, 2015), although it also infects monogastric animals and wild birds (Ayele et al., 2001; Pieper et al., 2014), causing chronic gastroenteritis with lymphangiectasia and lymphangitis; these lesions generate the appearance of the syndrome of bad nutrient absorption, weight loss, chronic or intermittent diarrhea, causing weakness and eventually death (Chiodini et al., 1984; Whittington et al., 2012; Mcgregor et al., 2015).

The fecal-oral pathway is the main transmission route for MAP (Whittington and Windsor, 2009), whether through lactation or by consumption of contaminated fodder (Chiodini et al., 1984; Windsor and Whittington, 2010). PTB has a chronic subclinical phase (Subharat et al., 2012), and factors such as stress, parasite, viral or bacterial infection, influence the transition from the subclinical status to the clinical status (Ayele et al., 2001), with an incubation period close to two years in small ruminants (Whittington et al., 2012).

Initially, PTB generates lesions that are restricted to the walls of the small intestine and mesenteric lymph nodes, but as the disease progresses, diffuse granulomatose inflammation is generated, with a variable number of acid-fast bacteria (AFB). Thus, the lesions are developed in the ileum, jejunum, terminal small intestine, cecum and colon (Clarke, 1997; Sigurðardottir et al., 2004). However, MAP can be disseminated to extra-intestinal sites, such as the reproductive tract and semen in bulls (Larsen and Kopecky, 1970; Larsen et al., 1981; Ayele et al., 2004; Khol et al., 2010), and ram semen (Eppleston and Whittington, 2001), although it has not yet been reported in reproductive tissue of sheep. However, the infected reproductive organs do not present an inflammatory response (Buergelt et al., 2004). And although no histological lesions have been observed in bovine testicle, the presence of MAP in this tissue has been determined, with the culture and polymerase chain reaction (PCR) technique (Glawischnig et al., 2004).

Therefore, the use of studs infected in reproductive tissue and semen represents a risk of MAP dissemination via horizontal sexual transmission because it is an extra-intestinal reservoir (Buergelt et al., 2004). Thus, the possibility of propagating PTB via semen is cause for great concern in the livestock production sector, because of economic losses and risks for public health, due to its relation to Crohn disease in humans (Ghadiali et al., 2004).

PTB has a global distribution and its prevalence ranges from 5 to 25% (Martínez-Covarrubias et al., 2012). In Mexico, PTB is widely distributed, with studies indicating that the rate of prevalence is between 5 and 30%, primarily in bovine, caprine and ovine livestock and fighting bulls (Guzmán-Ruiz et al., 2016). Chávez-Gris et al. (2004) reported prevalence of PTB in the states of Querétaro and Guanajuato, of 4.4% in ovine (*Ovies aries*) and 8.8% in caprine (*Capra hircus*) livestock, using the Restriction Fragment Length Polymorphism (RFLP) test. However, the subclinical nature of the infection and the scarce performance of diagnostic studies in flocks or herds in Mexico make this disease underestimated; this causes a negative impact in the economy of the ovine exports (Morón-Cedillo et al., 2013). In addition, in Mexico, sanitary control is not carried out in genetic improvement programs to certify that ovine semen is free of MAP, possibly because ovine PTB has received scarce attention. Nevertheless, PTB advances slowly in populations of small ruminants, unless management measures are introduced, since the accumulation of infection by MAP in the population will become ever-growing (Windsor, 2015). Therefore, the objective of this study was to identify the presence of MAP in reproductive tissue and semen of infected Pelibuey rams in clinical state of PTB.

## Materials and methods

### Study area

The experiment was carried out in observation and isolation pens that belong to the ovine flock of the Colegio de Postgraduados (COLPOS) Córdoba campus, research and study unit with records of PTB prevalence, which allows performing control and prevention protocols of sheep infected naturally. Located on the federal Córdoba-Veracruz highway, km 384, congregation Manuel León, Municipality of Amatlán de los Reyes, Veracruz, with geographic location at 18°51′20″ N and 96°51′37″ W at an altitude of 720 m, the climate is warm subhumid, with average temperature of 18 °C, with annual precipitation of 1,807.3 mm (García, 2004). The rams were housed in different facilities, in corrals separated by group, to prevent a possible infection, under the same conditions of intensive management. The experiment was designed under the criteria of the Mexican Official Norm (NOM-062-ZOO-1999) (Sagarpa, 1999) on technical specifications, for production, care and use of laboratory animals in agreement with the regulations for the use and care of research animals, approved by the General Academic Council of Colegio de Postgraduados, Mexico (Colpos, 2016). In addition, under the specifications of the Mexican Official Norm (NOM-033–ZOO–1995) (Sagarpa, 1996) for the sacrifice of animals used.

### Animals

Eight rams of Pelibuey breed were used, of which seven were diagnosed with PTB in a clinical state and an uninfected ram, which was used as negative control. The diagnosis trials were nested PCR (nPCR) and bacteriological culture, to detect MAP in feces; and enzyme-linked immunosorbent assay (ELISA) to detect antibodies. The average weight and age of the sheep was 57.23 ± 1.73 kg and 2.91 ± 0.17 years, respectively. All the rams had similar characteristics of management, age, weight and breed, also in order to ensure a similar degree of infection. The inclusion of an animal that is not infected was in order to corroborate the non-contamination of the samples, during the collection and their processing.

### Clinical signs

Table 1 shows the clinical signs at the time before the sample; of the seven rams infected, three presented cachexia (42.8%), five presented thick feces (71.4%) and only two presented diarrhea (28%). The seven infected rams presented emaciation (100%). At least two rams presented one of the signs mentioned (28%) and two rams presented all the signs indicated (28%). A ram was used as negative control (N).

**Table 1:** Clinical signs of PTB in Pelibuey rams infected naturally.

Ram identification	Clinical signs			
	Cachexia	Thick feces	Diarrhea	Emaciation
1	+	+	+	+
2	+	+	+	+
3	-	-	-	+
4	-	-	-	+
5	+	+	-	+
6	-	+	-	+
7	-	+	-	+
N	-	-	-	-

¨N¨ ram used as negative control, ¨+¨ positive observation, ¨-¨ negative observation, during the assessment of clinical signs of PTB.

### Sample collection

The blood samples (5 mL) were collected via jugular punction. Then, centrifugation was done (1000 × *g* for 10 min) to recover the serum in sterile 2 mL collecting tubes. Feces collection was carried out rectally with gloves and sterile collecting bag. Semen collection was done through artificial vagina, based on the protocol proposed by Bergstein-Galan et al. (2017). Before starting the semen collection, cutting of preputial hairs was done, and preputial washing was carried out with antiseptic and disinfectant liquid soap (Dermocleen®). One semen collection was used per ram, to carry out the culture and extraction of DNA. To perform tissue collection, the sacrifice of rams was carried out under humanitarian conditions, under specifications of the Mexican Official Norm (NOM–033–ZOO–1995), with an overdose of intravenous barbiturate (T61®, Intervet, S.A., Mexico). At the time of performing the tissue collection from each sample, surgical knife, forceps and new gloves were used to avoid crossed contamination. The tissue samples (20 g) were macerated in sterile conditions and placed in sterile collecting tubes of 50 mL. Finally, the storage of the samples of serum, feces, tissues (spleen, intestine, mesenteric lymph nodes, epididymis, Cowper gland, prostate, testicles), and semen was at -20 °C for their later processing in the laboratory of the National Center for Disciplinary Research-Animal Microbiology (*Centro Nacional de Investigación Disciplinaria*, CENID), of the National Forestry, Agricultural and Livestock Research (*Instituto Nacional de Investigaciones Forestales, Agrícolas y Pecuarias*, INIFAP).

### Serum analysis

The ELISA test was carried out to perform the determination of antibodies in serum samples, according to the methodology described by Martínez-Covarrubias et al. (2012). This trial has a sensitivity of 79.31% and a specificity of 82.25%, where the values higher than 0.194 optical densities were considered as positive.

### DNA extraction and nested PCR procedure

The DNA extraction was performed from samples of feces, tissues and semen, with the commercial kit, Kit BDtract^TM^ Genomic, using the manufacturer’s protocol. The conditions of the Master Mix and the nPCR amplification for all the samples were the same, according to the protocol by Martínez-Covarrubias et al. (2012), with primers for the sequence of insertion 900 (IS900), in the DNA samples of feces, tissues and semen. The primers for the first and second reaction were the ones recommended by Erume et al. (2001); for the first reaction: Paratb1 (5´-TGA TCT GGA CAA TGA CGG TTA CGG A-3´) and Paratb 4 (5´-CGC GGC ACG GCT CTT GTT-3´), with which a product of 563 pairs of bases (pb) was obtained. For the second reaction, the primers Paratb 2 (5´-GCC GCG CTG CTG GAG TTG A-3´) and Paratb 3 (5´-AGC GTC TTT GGC GTC GGT CTT G-3´) were used, with which a final product of 210 pb was obtained. DNA was obtained from a MAP strain (ATCC #700535) as positive control. The amplification product expected was visualized in agarose gel at 1.5% dyed with ethidium bromide. The electrophoresis was performed in a horizontal chamber model Horizon 11.14 (Life Technologies^®^). The conditions of electrophoresis were 45 min at 87 volts. The gel was observed in a transilluminator (model Gel Doc 2000, Marca Bio Rad^®^ of ultraviolet light) and the image was captured by computer with the Quatitvone software.

### MAP culture

The bacteriological isolation was carried out according to the protocol by Martínez-Covarrubias et al. (2012), where the tissue and semen samples were decontaminated with the alkali acid method and were sown by duplicate in a Herrorld cultivation medium, supplemented with egg yolk and mycobactin (2 mg L^-1^, Allied Monitor Inc.). The incubation was at 37 °C for a period of 14 weeks. To confirm the presence of AFB, a Ziehl-Neelsen (ZN) tincture was performed.

## Results

**Figure 1:**
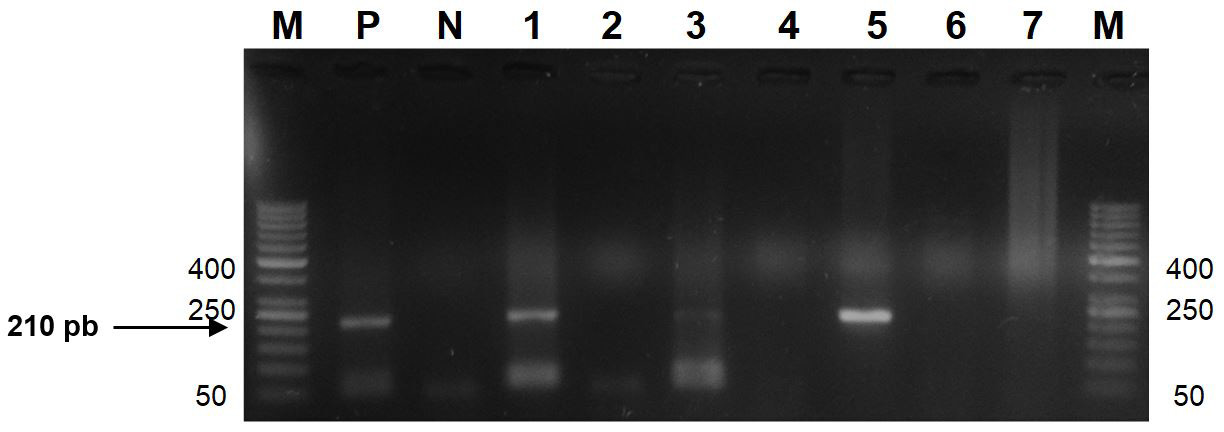
DNA amplification by nPCR in semen samples from infected rams with PTB in agarose gel electrophoresis (1.5%). Lanes: M: molecular marker 50 pb; P: positive control (MAP DNA MAP ATCC #700535); N: negative DNA control of ram semen; DNA samples from semen from animals included in the experiment: 1 (positive), 2 (negative), 3 (positive), 4 (negative), 5 (positive), 6 and 7 (negative).

**Figure 2:**
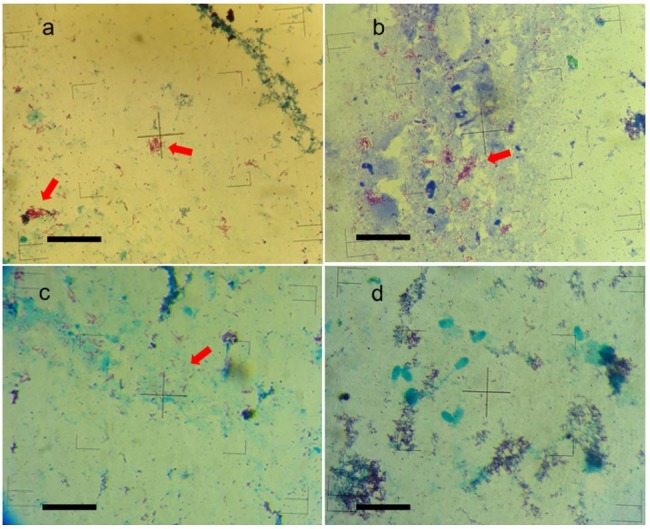
Ziehl-Neelsen (ZN) staining from the isolates obtained from the bacteria cultures of: a) intestine, b) mesenteric lymph nodes, c) testicle and d) semen. The arrows in red color indicate the presence of acid-fast bacteria (AFB). Bar scale = 10 μm.

The nPCR allowed identifying the presence of MAP in semen of infected rams, when visualizing a 210 pb band in amplified products (Figure 1). Of the DNA samples obtained from the semen, 42.9% (3/7) were positive in nPCR (Table 2). However, positive results were not obtained for MAP in semen with the culture technique (Figure 2d).

**Table 2:** Presence of *Mycobacterium avium* subsp. *paratuberculosis* in tissue and semen samples.

Identification of rams	Spleen		Intestine		Mesenteric lymph nodes		Epididymis		Cowper gland		Prostate		Testicles		Semen	
	nPCR	Culture	nPCR	Culture	nPCR	Culture	nPCR	Culture	nPCR	Culture	nPCR	Culture	nPCR	Culture	nPCR	Culture
N	-	-	-	-	-	-	-	-	-	-	-	-	-	-	-	-
1	+	+	+	+	+	+	+	-	+	-	-	-	+	+	+	-
2	-	-	-	-	-	-	-	-	-	-	-	-	-	-	-	-
3	-	-	+	+	+	+	-	-	+	-	+	-	+	-	+	-
4	-	-	-	-	-	-	-	-	-	-	-	-	-	-	-	-
5	-	+	-	+	+	+	-	-	-	-	-	-	-	+	+	-
6	-	-	+	+	-	-	-	-	-	-	-	-	-	-	-	-
7	-	-	-	-	-	-	-	-	-	-	-	-	-	-	-	-

“+” = positive result, “-” = negative result.

To determine the presence of AFB, the ZN staining was carried out (Figure 2) from the isolates obtained from the bacteriological cultures of digestive (a), lymphatic (b), reproductive (c) and semen (d) tissues. The staining showed 19.6% (11/56) of the presence of AFB in all the tissue types, but no MAP growth was shown in the semen samples (d).

The presence of MAP was identified in samples of spleen in one out of seven (14.3%) and two out of seven (28.6%), and small intestine in three out of seven (42.9%) and four out of seven (57.1%) tissue per nPCR and culture, respectively. For the case of the mesenteric lymph node tissue, the presence of the mycobacterium was found in three out of seven (42.9%) with both techniques. Likewise, it was identified in samples of reproductive tissue: one out of seven in epididymis (14.3%), two out of seven in Cowper gland (28.6%) and one out of seven in prostate (14.3%), with the nPCR test. However, in the cultures performed with these three reproductive tissues, no growth was observed in the mycobacterium. In the testicle samples, three out of seven were identified as infected with MAP (42.8%; culture or nPCR technique), but only in two out of seven (28.6%) had MAP presence obtained with both techniques (Table 2).

## Discussion

In the revision of the infected rams, signs were found that belong to PTB; emaciation in the seven rams (100%) and thick feces in five out of the seven infected rams (71.4%). It has been reported that the presence of these signs are due to the diffuse hypertrophy caused in the mucosa of the jejunum and ileum, which provokes macroscopic thickening of the intestinal wall; these granulomatous lesions are caused by MAP and generate bad absorption of nutrients, provoking loss in body condition (Clarke, 1997; Verin et al., 2016). The establishment of MAP in the small intestine should be expected, especially in the lamina propria and submucosa, as well as in the capsule and cortex of the lymph nodes associated to the intestine (Larsen et al., 1981; Whittington et al., 2012; García and Shalloo, 2015). Thus, in this study, it is indicated that the samples from the small intestine presented the highest number of positive results (four out of seven in culture and three out of seven with nPCR). However, in this study’s rams the thickening of the intestinal walls was not very evident, making difficult the localization of the granulomatous lesions. This had an effect on the results, since not all the rams were positive to the localization tests and the identification of MAP in the intestine (ileum). This is possibly due to the type of MAP strain, since it has been reported that the lambs infected with Type C strains presented a higher inflammatory response, while in those infected with the Type S strain, the lesions were minimal (Fernández et al., 2014).

This result confirms that the intestinal tract is the main site of PTB infection; however, MAP has also been isolated in extra-intestinal sites (Hasonova et al., 2009), such as the identification in this study of MAP by nPCR one out of seven (14.3%) and by culture two out of seven (28.6%) in spleen. For the case of the mesenteric lymph node samples of this study, MAP was identified in three out of seven (42.9%) with both tests. The latter value presented a similarity with what was reported by Hasonova et al. (2009) and Hemalatha et al. (2013), who found values of 61.2% and 53%, respectively. This is due mainly to the fact that lymph nodes and the small intestine are the tissues where the lesions primarily develop, as confirmed by Eppleston and Whittington (2001), who reported the MAP culture from samples of inguinal eight out of eleven (72%) and ileum eleven out of eleven (100%) lymph nodes in lambs with clinical status of PTB. It is relevant to emphasize that the presence of MAP in extra-intestinal sites is possibly due to the diffusion through blood and the lymphatic system (Buergelt et al., 2004), allowing to find the mycobacterium in other tissues different from the intestinal.

Therefore, MAP can be identified in other extra-intestinal sites, such as the seminal vesicle and prostate (Larsen et al., 1981), in testicular tissue (Glawischnig et al., 2004) and bovine semen (Larsen et al., 1981; Khol et al., 2010). In this study, the nPCR test detected MAP in tissue samples of epididymis, Cowper gland and prostate; however, the culture technique did not allow detecting positive results of MAP in these tissues. This indicates that the nPCR technique was more sensitive than the culture technique. According to what was pointed out by Nebbia et al. (2006), who state that MAP culture is a diagnosis tool that is not sensitive, particularly when mycobacteria are no longer viable, in comparison to the molecular techniques which can contribute to the identification of the etiological agent even when it is not viable. Likewise, it has been reported that the culture is capable of offering an adequate diagnosis starting from 10^10^ viable organisms of the S strain of MAP, in sheep (Windsor, 2015). Meanwhile, Whipple et al. (1992) reported that the PCR with IS900 primers is capable of detecting infections with just 10^4^ colony forming units (CFU) per gram, in bovine feces.

In the samples of testicle tissue, two out of seven (28.6%) of tissue infected by MAP was identified with the nPCR and culture tests. These techniques have also revealed the presence of the mycobacterium in testicle tissues from a bovine in clinical state, despite the tissues not showing clinical lesions; these results exhibit the possibility of MAP transmission in semen (Glawischnig et al., 2004). Similarly, Larsen et al. (1981) and Ayele et al. (2001) described that bulls diagnosed in clinical state can be infected in several parts of their reproductive organs and, as consequence, can carry the bacteria in semen, which could convert the semen into a vector of horizontal transmission. Likewise, in sheep in clinical state of PTB infection, Eppleston and Whittington (2001) obtained positive isolates in ovine semen three out of eleven (27%), confirming that the bacteria has the capability of infecting semen, although they did not define the origin of MAP. However, in contrast with this study, the authors did not report positive results in reproductive organs. This difference in the results obtained can be due to the amount of viable CFUs present in the tissue and semen samples. Additionally, this is the first study that confirms the presence of MAP with nPCR in epididymis, Cowper gland, prostate and testicles of hair sheep in the clinical state of the PTB infection. Sharifzadeh et al. (2010) have discussed that the presence of MAP in semen is possibly due to the ability of the organism to survive to antibiotic treatments; in addition, they emphasize the property of MAP to resist cryopreservation in liquid nitrogen. In this study the presence of MAP was determined with nPCR in 42.9% (3/7) of the semen samples; however, the bacteria could not be cultured in any of the semen samples. This result agrees with what was reported by Khol et al. (2010), who reported the presence of MAP in all the semen samples of a bovine with PCR and IS900 primers, but did not report the isolation of the mycobacterium in culture. As the authors mention, this is possibly because of the low number of bacteria present in the semen or their low viability, caused by the process of decontamination of the sample. On the other hand, it has been reported that the process of freezing and thawing of the samples can contribute to the decrease in the viability of the mycobacteria and, consequently, decrease the subsequent positive result of MAP in culture (Martínez-Covarrubias et al., 2012). However, although the mycobacteria from the semen samples used in this study did not show the capability of replication in culture, the identification of MAP in semen through nPCR does reveal the potential risk of horizontal transmission to the female via semen, which has already been considered in bovines (Larsen et al., 1981; Ayele et al., 2004). To test the risk of horizontal transmission, cows have been inseminated with doses of 5×10^8^ CFU of MAP, twenty four hours after artificial insemination or after natural mounting; then this mycobacterium was isolated in uterus and in uterine horns during the first week, in utero and pelvic lymph nodes in the second week (Perry et al., 2006). For the area of animal reproduction, the identification of MAP in reproductive tissue and semen is of great relevance, since this study confirms the presence of MAP in tissues and organs that are not considered target organs, in addition to the fact that infected rams can be a source of infection.

On the other hand, it should be considered that this type of infection could delay the disease control programs (Whittington and Windsor, 2009). Therefore, it is recommended to implement diagnosis measures and timely management, to identify and eliminate the rams that may be the cause of propagation or permanence of PTB in the flocks where it is necessary. In our laboratory, no technique was implemented to guarantee the elimination of MAP in semen, because at the time of the study it only suggested identifying the presence of MAP in semen; however, Bielanski (2012) recommends implementing a gradient of centrifugation and/or swim-up, since it has been proven that this procedure decreases the risk of transmitting diseases in receptor females or *in vitro* fertilization systems. Additionally, it is convenient to perform the processing of semen according to the standards of the International Embryo Technology Society (IETS) (Stringfellow and Givens, 2010).

In general, Wentink et al. (2000) recommend performing serological tests with a random sample of 20%, with the aim of ensuring the absence of the infectious agents in the flock or herd. On the other hand, it is recommended to implement routine hygiene and cleaning procedures, since it is important to minimize the exposure to manure, which is where the causal agent is found. In addition, it is suggested to perform the PCR technique in semen, with the aim of identifying MAP and avoiding the propagation of this pathogen agent with the use of semen via artificial insemination, since, as has been mentioned before, this mycobacterium can resist treatments with antibiotics and cryopreservation processes. This study allowed identifying the presence of *Mycobacterium avium* subsp. *paratuberculosis* in lymphatic tissue, digestive tract, and semen and, for the first time, in epididymis tissue, Cowper gland, prostate and testicles of naturally infected Pelibuey rams.
